# Sex-Related Differences in Photoplethysmography Signals Measured From Finger and Toe

**DOI:** 10.1109/JTEHM.2019.2938506

**Published:** 2019-08-30

**Authors:** Seyedmohsen Dehghanojamahalleh, Mehmet Kaya

**Affiliations:** Department of Biomedical and Chemical Engineering and SciencesFlorida Institute of Technology 5401 MelbourneFL32901USA

**Keywords:** Sex, PPG, finger, Toe, PTT

## Abstract

Sex plays an important role in the normal cardiovascular system function including resting heart rate and arterial blood pressure. In addition, it has been reported that men and women are at different levels of risk for cardiovascular diseases. The aim of this study was to evaluate and compare the temporal and morphological features of both finger and toe photoplethysmography (PPG), and anthropometric and biological parameters with respect to sex. A customized PPG and electrocardiography (ECG) combo device was developed to measure the signals of interest. ECG/PPG features in addition to subjects’ information were compared regarding finger and toe PPGs. Eighty-eight subjects participated in the study. Linear regression and Student’s t-test were used for statistical analysis. Our results revealed that pulse arrival time (PAT), pulse transit time (PTT), systolic pulse transit time (SPTT), and the ratio of areas under the PPG waveform from the onset to the inflection point and the inflection point to the end of the waveform (S2/S1), are dependent on sex. The highest dependence was shown for the finger PTT while the toe PTT did not indicate any significant dependence on sex. This is the first study that evaluates the effect of sex on cardiovascular system function using finger and toe PPG based features which can help to understand sex-based risk factors for cardiovascular diseases and to improve related disease management and treatments.

## Graphical abstract

The aim of this study was to compare, using a customized photoplethysmography (PPG)/ECG device, the temporal and morphological features of finger and toe photoplethysmography (PPG) and anthropometric and biological parameters with respect to sex. Our results in 48 subjects revealed that pulse arrival time (PAT), pulse transit time (PTT), systolic pulse transit time (SPTT), and the ratio of areas under the PPG waveform from the onset to the inflection point and the inflection point to the end of the waveform (S2/S1) are dependent on sex. These preliminary results can help to understand sex-based risk factors for cardiovascular diseases and may improve related disease management and treatment.

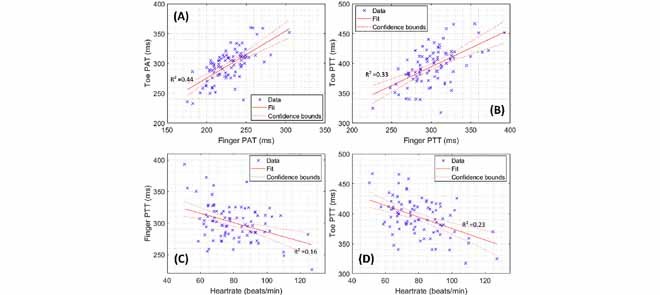

## I. Introduction

It has been shown that sex plays an important role in relation to certain diseases [1]. An increasing number of studies have indicated that cardiovascular functions such as arterial blood pressure and resting heart rate can vary between men and women. In addition, it has been reported that men have greater risk levels for cardiovascular diseases compared to pre-menopausal women [2]. This risk factor is valid for many cardiovascular diseases such as hypertension and coronary artery diseases. Therefore, it is essential to understand the contributing physiological factors leading to the mentioned differences in terms of sex. This study is the first to analyze and compare the differences in the finger and toe PPG based features in relation to sex.

Photoplethysmography (PPG) is the most common technique that is used to measure the peripheral blood volume as the indicator of blood pulse. This is performed by emitting a light beam (i.e. red, infra-red, or green) to the tissue and converting the reflected/transmitted light to an electrical signal [3]. A PPG signal consists of periodic cycles and the frequency of the repetition is equal to the heart rate (HR). Several body points can be used as the PPG signal recording site such as the fingertips, wrists, toes, earlobes, and forehead [4]–[9]. The pressure wave changes in shape and velocity depending on the stiffness and diameter of the artery and the viscosity of the blood.

**FIGURE 1.**
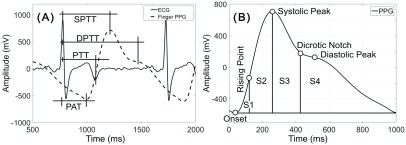
(A): PAT, PTT, SPTT, and diastolic peak transit time (DPTT) latencies measured from the R peak of the ECG signal; (B): The area under each PPG cycle are divided into four areas (S1, S2, S3, & S4). These areas are enclosed between the onset, systolic peak, dicrotic notch, and the end of the PPG cycle. The summation of S1-S3 finds A1, and A2 is equal to S4.

ECG is the measurement of the electrical activity of the heart over the body surface. ECG is a periodic signal that consists of waves that refer to specific events of the corresponding cardiac cycle; for example, ECG’s R peak is accepted as the beginning of the ventricular contraction. The time delay between an R peak and the onset of the consecutive PPG cycle is called the pulse arrival time (PAT); the time delay between an R peak and the point that the blood flow is at its fastest is called the pulse transit time (PTT); and the time delay between an R peak and the maximum point of the PPG signal is called the systolic pulse transit time (SPTT) (Fig. 1A). These delays are being used in applications such as continuous non-invasive blood pressure (CNIBP) monitoring, measurement of respiratory effort, pulse wave velocity (PWV) analysis, detection of microarousals, etc. [10], [11]. For example, in PWV analysis, the velocity is calculated by dividing the distance between the heart and the recording site by the PTT. The PWV is measured to assess the arterial stiffness and is a highly reliable prognostic parameter for cardiovascular morbidity and mortality. It has been shown that stiffer and subsequently less elastic arteries display shorter PTT [4], [6], [12], [13].

In order to perform a thorough analysis of the PPG signal, the dependence of physiological parameters (weight, height, BMI, age, and sex), temporal and morphological features (PTT, PAT, SPTT, S2/S1 ratio, and inflection point area (IPA)), and cardiovascular information (heart rate and variance of heart rate) and their effect on one another was investigated.

Moreover, this comparison was performed on both finger and toe PPG signals in order to identify the parameters which had the highest dependency on these recording sites.

The findings from this study can be used in several applications including CNIBP, PWV and arterial compliance analyses for more accurate and efficient computations and estimations.

## II. Methods and Procedure

In this study, a measurement device was built to amplify and capture the signals. The main parts of this device are a 3-input amplifier and a data handler module which works as a central processing and control unit. The amplifier amplifies PPG signals from two different spots and an ECG signal. Signals were filtered considering their bandwidths. The PPG signals were conditioned by a 2^nd^ order high pass filter at 0.5 Hz and a 6^th^ order low pass filter at 25 Hz. The ECG signal was filtered using a 2^nd^ order high pass filter at 0.5 Hz and a 6^th^ order low pass filter at 100 Hz. Analog-to-digital conversions (10-bit, 1 kHz) and data flow control were performed using an ARM Cortex-M4 (Tiva Launchpad TM4C123G, Texas Instruments, Dallas, TX, USA) evaluation board. A custom I/O board was also designed and printed, and the two mentioned units were mounted on it. The signals were simultaneously amplified and captured. A DC-DC converter was utilized to protect the circuit against the power supply input ripple noise and to provide a voltage barrier. A Bluetooth link was provided to transfer the recorded data to a PC at a speed of 115200 bits per second.

### A. Subjects

A total number of 88 recordings were obtained from 80 participants including 39 female and 41 male subjects with average ages of 24.1±8.0 and 25.8±7.2, respectively. Eleven subjects indicated to be smokers (8 males and 3 females). Six subjects (3 males and 3 females) indicated that they had hypertension. Written informed consents were obtained from all subjects. A digital questionnaire was completed by each subject that obtained general medical and anthropometric information of subjects. All the testing procedures with human subjects and the recording place were approved by the Office of Compliance and Risk Management Institutional Review Board (IRB Approval Number: 18-030) at Florida Institute of Technology, Melbourne, FL, USA. This study was conducted according to the Declaration of Helsinki ethical principles for medical research on human subjects.

### B. Features

Extracted features and a questionnaire were categorized into two main classes. The first class is based on ECG and PPG signals, and the second class is based on subject information:

• ECG/PPG based:
  a. Heart rate features,
  b. Latency/time features,
  c. Morphology features.
• Subject information-based features.

### C. Features

Heart rate: ECG signal was recorded for three minutes and the QRS complexes were detected. The time difference between every two consecutive R peaks is called the R-R interval. Instantaneous heart rate is calculated by dividing 60 by beat-to-beat R-R interval. The average heart rate (HR) and the variance of the heart rate (VHR) were used as heart rate features.

Latency/Time: When the blood reaches the lateral PPG location, the blood volume swiftly changes. PPG signal, as the indicator of blood volume, comprises of consecutive peaks, inflection points, and a notch, which indicate some of the features within the cardiovascular system (Fig. 1B) [14]–[16]. These points make the extraction of the latency/time features from the PPG signal possible. The primary peak is called the systolic peak and the second one is known as the diastolic peak [17], [18]. The time, when the blood volume begins to increase, is the onset of the PPG signal [18]. At this time, the first derivative of the signal becomes positive [14]–[18]. The time delay between the ECG R peak and the onset of the PPG signal is considered as PAT (Fig. 1A). It is shown that when the first derivative of the PPG signal is at its highest, the change in blood volume is at its maximum [17], [18]. This point is known as the rising point. The time delay from the ECG R peak to this moment is called PTT. SPTT and DPTT are known as the time delay between an R peak, and the systolic and diastolic peaks respectively (Fig. 1B) [14]–[18].

Morphology features: Each PPG cycle can be divided into two parts where the first part is from the beginning of the cycle until the dicrotic notch (A1) and the other one is from the dicrotic notch to end of the cycle (A2). The A2/A1 ratio is called IPA [16], [18], [19]. The total peripheral resistance is related to the A2/A1 ratio [18]. PPG cycles can also be demonstrated by 4 areas known as S1, S2, S3, and S4 (Fig. 1B) [16], [18], [19]. The most important feature that can be extracted from S1-S4 is the S2/S1 ratio that represents the peripheral resistance [18].

### D. Subject Information

Physiological and anthropometrical features such as weight, height, and sex were gathered from the questionnaire that the subjects filled out. BMI was calculated by dividing the subject’s weight (kg) by their squared height (m^2^) [20].

### E. Statistical Analysis

Two statistical approaches were employed in this study; Student’s t-test and linear regression.

The significance of each comparison was validated using student t-test. If the measured p-value using Student’s t-distribution was lower than the significance level ($\alpha$), the null hypothesis was rejected and the alternative hypothesis was accepted.

The relationship between the features was modeled using linear regression analysis (LRA). LRA is a linear method that fits a set of data on another one. The model uses a 1^st^ order linear function. For example, given two sets of data such as PTT: [ptt1, ptt2,…, pttn] and HR: [hr1, hr2,…, hrn] gathered from n subjects, LRA calculates a linear relationship between these sets as PTT = I + r $\times $ HR. Where I is the intercept and r is the slope and is also considered as the correlation coefficient.

The criteria of acceptance as statistical significance in this study were the p-values being lower than 0.05 and the r^2^ values being greater than 0.11 ($\vert \text{r}~\vert >0.33$); where $\alpha $ was set at 0.05.

### F. ECG Complex Detection

ECG QRS complexes were detected using equation (1) that is based on the derivatives of the ECG signal (u[n]). This detection technique was first introduced by N. M. Arzeno et al. [21].

\begin{equation*} V[n]=\frac {1}{8}(2u[n]+u[n-1]-u[n-3]-\;2u[n-4])\tag{1}\end{equation*}

where n is the sample number and $V$[n] is the filtered signal. Equation (1) was used to distinguish the peaks and also to prevent the effects of high-frequency noise features on the output. In the next step, the filtered signal was squared. Then, a 32-sample moving average filter made the filtered signal smoother. Finally, an adaptive threshold value detected the QRS complex. The middle point of the area above the threshold level was annotated as the location of the R peak.

## III. Results

### A. Latencies

The first step in our analysis was to compare the PTT and PAT values calculated from the finger and toe PPG signals. Since the distance from the heart to the toe is longer than the distance to the finger [4], a longer PTT and PAT would be observed from the toe than the finger PPG signals. Therefore, PTTs and PATs were normalized by the subjects’ height.

**FIGURE 2.**
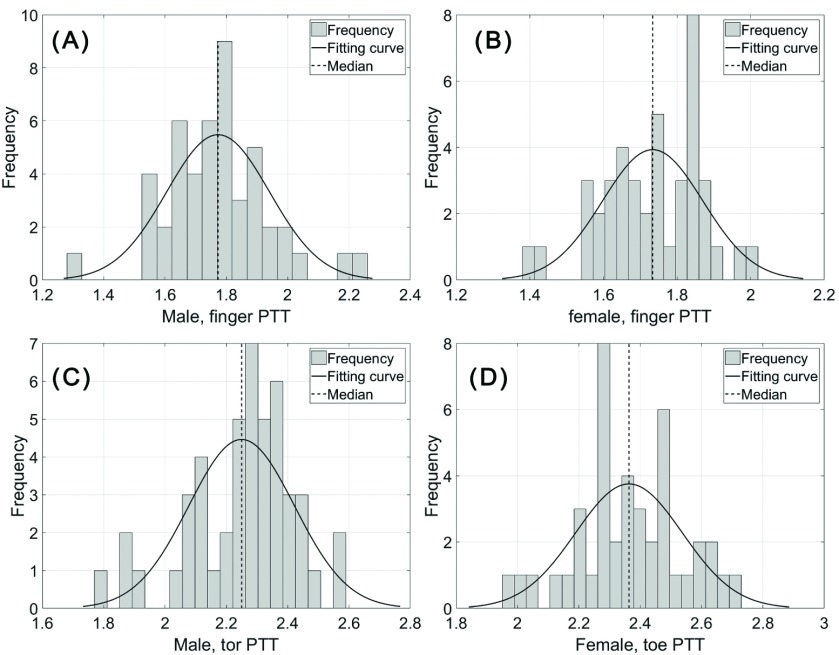
Distribution of PWV values, calculated for finger and toe PPGs: finger PTT of male subjects (a); finger PTT of female subjects (b); toe PTT of male subjects (c); toe PTT of female subjects (d).

The probability distribution of PTT in male and female subjects are displayed in Fig. 2 which shows that the observed parameters had normal distributions.

The mean, variance, and standard deviation (SD) values of the mentioned latencies for males, females, and both sexes are shown in Table 1.

**TABLE 1:** The Statistical Properties of the Finger and Toe PAT/PTT

		Mean	SD
Finger PTT (ms)	Male	313	28
	Female	287	22
	All	302	28
Toe PTT (ms)	Male	398	29
	Female	392	32
	All	397	31
Finger PAT (ms)	Male	237	22
	Female	217	19
	All	228	22
Toe PAT (ms)	Male	299	29
	Female	295	24
	All	297	26

### B. Sex

**FIGURE 3.**
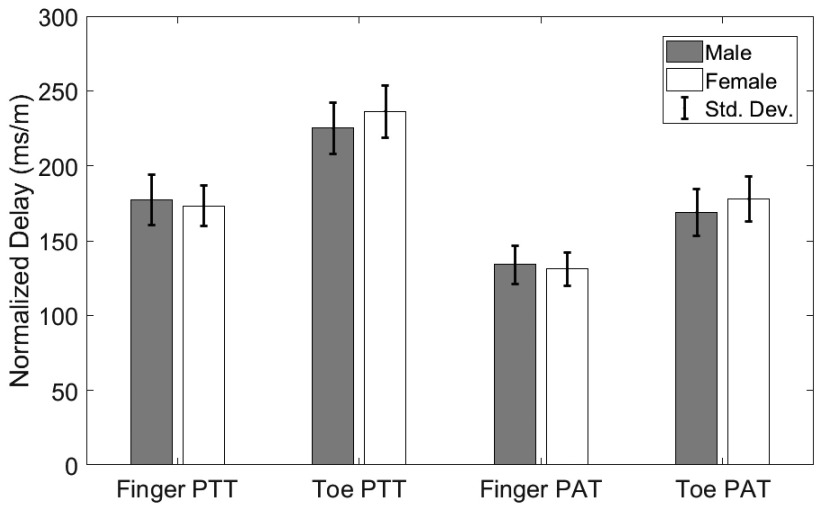
Mean and standard deviation of normalized pulse latency with respect to sex for finger PAT, finger PTT, toe PAT, and toe PTT.

Both PAT and PTT, either from the finger or toe, were greater in male subjects than in female subjects, which is a result of higher values of average height in men. Therefore, the delays are normalized by first dividing them by the subject height. The difference between the two sexes for normalized finger and toe PAT and PTT is shown in Fig. 3. As illustrated in this figure, males had greater normalized latencies than females.

### C. Height

Taller subjects have longer distances between their hearts and recording sites [4]. So, the latency between the QRS complex and the PPG peak was greater in taller subjects. It was calculated that the subject’s height affects PAT (toe: r = 0.38, p-value < 0.0001; finger: r = 0.46, p-value < 0.0001) and PTT (toe: r = 0.42, p-value < 0.0001; finger: r = 0.44, p-value < 0.0001).

### D. Blood Pressure

PTT is a function of BP and some subject-specific parameters that are the outcome of the long-term and short-term physiological and physical condition of each subject and differs from subject to subject [16], [19]. Therefore, although PTT and BP follow a specific trajectory in *one* subject, it cannot be considered as a dependency that can be generalized for every subject.

### E. Heart Rate

**FIGURE 4.**
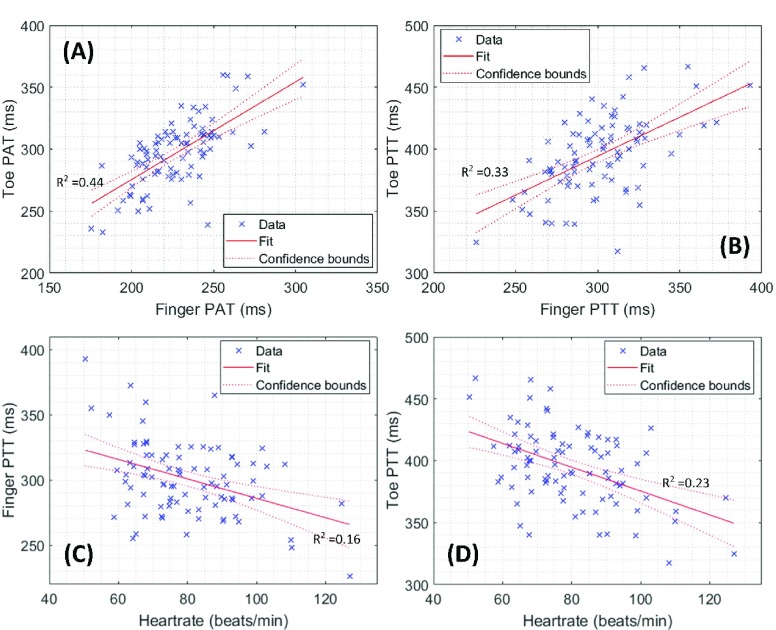
Comparing the dependence of finger and toe PAT (A) and PTT (B) on each other and the dependence of heart rate on PTT from finger (C) and toe (D). The linear regression lines are shown in solid line; the confidence interval of 95% is represented by the confidence bounds (dotted line).

HR is an indicator of cardiac activity. Higher heart rates increase the cardiac output, which is the multiplication of heart rate and stroke volume. Fig. 4 C and D show that the HR has a correlation with the toe PTT (r = -0.37, p-value < 0.001) and the finger PTT (r = -0.42, p-value < 0.001). It is also illustrated that the effect of HR on DPTT was more than PTT (r = -0.54, p-value < 0.001).

The effect of the recording site on PTT and PAT: The dependency of the finger and toe PAT/PTT on each other was also analyzed. The results showed that finger and toe PAT and PTT were highly correlated (PAT: r = 0.69, p-value < 0.0001; PTT: r = 0.59, p-value < 0.0001) and the finger and toe PAT had an even higher correlation than their PTTs. Fig. 4 A and B illustrate the dependence of finger and toe PTT and PAT on each other.

The PPG peak detection performance was evaluated by an expert and due to low detection accuracy of the diastolic peak in toe PPG, all the extracted features from this point were removed from the database.

The main assessed parameters in this study were PTT, PAT, SPTT, and S2/S1. These parameters were compared with all the other parameters; then the square of the correlation coefficient (r^2^), and p-values were calculated. The comparison results are presented in Table 2. Parameters such as sex, height, HR, and IPA had the highest dependency on PAT, PTT, SPTT, and S2/S1 (the dependency criteria were $\vert \text{r}\vert >0.33$ and p-value < 0.05).

**TABLE 2:** Comparing the Effect of SBP, DBP, Height, Weight, BMI, HR, VHR, Gender, and IPA on PTT, PAT, SPTT, and S2/S1. The Comparisons are Performed Using Linear Regression Analysis $\alpha=0.05$)

	Finger			Toe		
Parameter	slope	$r^{2}$	p-value	slope	$r^{2}$	p-value
PTT						
SBP	0.04	–	0.841	−0.24	0.0117	0.33
DBP	0.02	–	0.956	−0.92	0.076	0.011
Height	1.34	0.195	<1e−5	1.36	0.184	<1e−5
Weight	0.26	0.030	0.116	0.19	0.015	0.276
BMI	−0.13	−0.011	0.821	−0.45	−0.007	0.438
HR	−0.73	0.142	<1e−5	−0.86	0.181	<1e−5
VHR	−0.20	0.030	0.114	−0.25	0.046	0.05
Gender	27.56	0.242	<1e−5	8.09	0.019	0.213
IPA (finger)	35.07	0.047	0.045	51.39	0.092	0.005
PAT						
SBP	−0.02	–	0.993	−0.17	0.008	0.413
DBP	−0.27	–	0.339	−0.73	0.073	0.013
Height	1.07	0.214	<1e−5	1.02	0.146	<1e−5
Weight	0.18	0.024	0.16	0.15	0.013	0.29
BMI	−0.24	0.003	0.587	−0.30	0.005	0.539
HR	−0.59	0.148	0.0003	−0.68	0.158	0.0002
VHR	−0.15	0.029	0.125	−0.24	0.058	0.029
Gender	20.66	0.219	<1e−5	6.47	0.017	0.239
IPA (finger)	43.83	0.119	0.001	51.09	0.128	0.0009
SPTT						
SBP	0.02	–	0.944	−0.28	0.011	0.355
DBP	0.56	0.016	0.257	−0.95	0.054	0.033
Height	1.29	0.094	0.004	1.10	0.080	0.009
Weight	0.13	0.004	0.572	0.05	0.001	0.793
BMI	−0.54	0.007	0.483	−0.73	0.013	0.307
HR	−0.99	0.137	0.0005	−1.16	0.216	<1e−5
VHR	−0.24	0.023	0.164	−0.28	0.039	0.074
Gender	29.43	0.144	0.0004	4.94	0.005	0.536
IPA (finger)	20.63	0.009	0.407	59.55	0.083	0.008
S2/S1						
SBP	−0.02	0.028	0.129	0.003	0.004	0.569
DBP	0.002	0.0001	0.923	0.012	0.021	0.188
Height	−0.04	0.061	0.024	−0.03	0.151	0.0003
Weight	−0.02	0.085	0.073	−0.007	0.032	0.104
BMI	−0.05	0.041	0.065	0.0003	–	0.981
HR	−0.01	0.016	0.25	−0.008	0.023	0.169
VHR	0.0006	–	0.934	−0.001	0.001	0.774
Gender	−1.12	0.126	0.001	−0.24	0.028	0.129
IPA (finger)	−0.22	0.0005	0.83	0.17	0.001	0.714

Finger PTT had higher dependency on the subject’s height (r^2^ = 0.2, p-value < 0.0001) than toe PTT (r^2^ = 0.18, p-value < 0.0001) while HR showed a stronger dependence on toe PTT (r^2^ = 0.18, p-value < 0.0001) than finger PTT (r^2^ = 0.14, p-value < 0.0001). In addition, sex only affects finger PTT (r^2^ = 0.24, p-value < 0.0001) and had no correlation to toe PTT.

Very similar to PTT, finger PAT’s dependency on height (r^2^ = 0.21, p-value < 0.0001) was higher than toe PAT (r^2^ = 0.15, p-value < 0.0001) and HR was more effective on toe PAT (r^2^ = 0.15, p-value < 0.001) than finger PAT (r^2^ = 0.15, p-value < 0.0001). Moreover, toe IPA indicated greater r^2^ and lower p-value for toe PTT (r^2^ = 0.13, p-value < 0.001) than finger PTT (r^2^ = 0.12, p-value < 0.01). And finally, sex showed no relationship to toe PTT but was related to finger PTT (r^2^ = 0.22, p-value < 0.0001).

Both finger and toe SPTT were related to HR but toe SPTT had a stronger relationship (r^2^ = 0.22, p-value < 0.0001) than finger SPTT (r^2^ = 0.14, p-value < 0.001). Sex showed no dependency on toe SPTT while it displayed a relationship to finger SPTT (r^2^ = 0.14, p-value < 0.001).

Height and sex were the only two parameters that had dependency on toe S2/S1 ratio (r^2^ = 0.15, p-value < 0.0003) and finger S2/S1 ratio (r^2^ = 0.13, p-value < 0.001), respectively. The other parameters showed no statistical significance.

## IV. Discussion

In this study, a comprehensive set of comparisons was performed between the extracted parameters from the finger and toe PPG signals. The major benefit of this comparison is to assess the effect of sex and other factors on the toe and finger PPG signals.

Even though the previous studies evaluated the effect of parameters such as age, systolic blood pressure, height, and HR on features (primarily PTT) extracted from ear, finger and toe PPGs [4]–[6], [22], [23], they did not analyze additional important physiological parameters such as weight, sex, BMI, or morphological parameters including IPA, and VHR. The major contribution of this study is to integrate these additional parameters and provide insights regarding the impact of these features on both the toe and finger PPG signals. Including these parameters can help improve future studies related to PWV analysis, CNIBP, BP variability analysis, etc.

As shown in Table 2, PAT, PTT, SPTT, and S2/S1 had higher dependencies on sex, height, HR, and IPA. Due to the structural differences between the vascular pathways that end at the PPG recording sites, the location of the sensor can affect the revealed information. For example, our results show that sex plays an important role in the latencies and the morphology of the finger PPG, while the toe PPG was not affected similarly.

### A. Heart Rate

HR is an indicator of the sinusoidal node rhythm and the autonomic nervous system activity. In a study by M. J. Drinnan et al., it was shown that there is a stronger correlation between HR and finger PTT [24]. Similarly, our study also showed a high dependency of HR on finger PTT. However, other time features (PAT and SPTT) and other PPG sites other than the finger were not included in their study [17]. Our results demonstrated the HR correlation to PTT and indicated HR’s dependency on PAT and SPTT as well: PAT and SPTT, either from finger or toe (PTT_finger_: r^2^ = 0.14, p-value < 0.001, PAT_finger_: r^2^ = 0.15, p-value < 0.001, SPTT_finger_: r^2^ = 0.14, p-value < 0.001, PTT_toe_: r^2^ = 0.18, p-value < 0.001, PAT_toe_: r^2^ = 0.16, p-value < 0.001, SPTT_toe_: r^2^ = 0.22, p-value < 0.001). Therefore, in applications such as CNIBP, where peripheral pulse latencies are the major features of concern, HR must also be included in the features matrix.

### B. Sex

Pulse wave propagation delay is a function of the stiffness of the vasculature [25]. Hence, stiffer arteries display shorter pulse propagation latencies [25]. In a couple of studies, the dependency of sex on arterial stiffness is assessed and it was inferred that women had relatively stiffer arteries than men [26], [27]. Our statistical analysis showed that sex had correlations with some of the parameters extracted from finger PPG (PTT_finger_: r^2^ = 0.24, p-value < 0.001, PAT_finger_: r^2^ = 0.22, p-value < 0.001, SPTT_finger_: r^2^ = 0.14, p-value < 0.001, S2/$\text{S}1_{\mathrm {finger}}$: r^2^ = 0.13, p-value = 0.001) while its dependence on the toe PPG was *not* statistically significant. Our results supported the previous studies, but they additionally showed that sex and arterial stiffness do not make any significant temporal differences in the PPG signal from the lower limbs. Therefore, using/including toe PPG in an application helps to suppress the effect of sex difference on peripheral latencies. In contrast, finger PPG can be employed in applications that assess the dependence of sex on arterial stiffness or peripheral latencies.

### C. Height

As expected, the pulse latencies were longer in taller subjects than the shorter ones. In contrast, SPTT was not significantly correlated to the height (PTT_finger_: r^2^ = 0.20, p-value < 0.001, PAT_finger_: r^2^ = 0.21, p-value < 0.001, PTT_toe_: r^2^ = 0.18, p-value < 0.001, PAT_toe_: r^2^ = 0.15, p-value < 0.001). Besides, height and toe S2/S1 also displayed dependency (r^2^ = 0.15, p-value < 0.001) while finger S2/S1 had a low correlation to the height. Therefore, in applications such as CNIBP that are based on peripheral pulse latency, the time delays must be normalized by dividing them by the subject height.

One of the limitations of this study was the number of subjects. For our future study, we are planning to increase the number of PPG sensors and subjects. The goal of adding new sensors is to further assess the dependence of the parameters on different parts of the body. Another limitation can be the subjects that indicated they were smokers (8 males and 3 females) and the subjects who had hypertension even though they were evenly distributed (3 males and 3 females). However, the number of those subjects were relatively low to perform a separate and reliable analysis.

The findings in this study can be used in several applications. First, in applications that are based on peripheral pulse latency, it is crucial to normalize the time delays by dividing them by the subject height. Second, since some of the parameters are correlated, the parameters with high correlation can be fused together (i.e. PAT and PTT) or be decreased in number to reduce the dimension of the features matrix used in machine learning algorithms. Thirdly, including toe PPG in an application such as CNIBP can reduce the effect of sex on the results while adding finger PPG is a good strategy when the effect of sex on the cardiovascular system is studied (i.e. PWV analysis).

## V. Conclusion

To conclude, sex was correlated to the parameters extracted from the finger PPG (PAT, PTT, SPTT, and S2/S1) but showed no dependence on any of the parameters from the toe PPG. The analysis and comparison of the temporal (i.e. IPA and S2/S1) and morphological (i.e. PAT and PTT) features from the finger and toe as well as the subjects’ general data revealed important information and differences in the PPG signals which can be due to different arterial structures leading to those sites. Furthermore, HR, sex, and height were shown to be the major parameters that affect the PPG parameters. It was shown that two of the pulse latency parameters (PTT and PAT) were highly correlated to HR and the subject’s height.

These findings can be used in several applications including CNIBP, PWV and arterial compliance analyses for more accurate and efficient computations and estimations.
